# Using Network Pharmacology to Systematically Decipher the Potential Mechanisms of Jisuikang in the Treatment of Spinal Cord Injury

**DOI:** 10.1155/2022/4932153

**Published:** 2022-02-27

**Authors:** Shaoshuo Li, Yang Shao, Hao Chen, Jianwei Wang

**Affiliations:** ^1^Nanjing University of Chinese Medicine, Nanjing 210023, China; ^2^Department of Traumatology and Orthopedics, Wuxi Affiliated Hospital of Nanjing University of Chinese Medicine, Wuxi 214071, China

## Abstract

**Objective:**

To identify the potential pharmacological targets of Jisuikang (JSK) for the treatment of spinal cord injury (SCI) using network pharmacology.

**Methods:**

The bioactive compounds of JSK herbs and their corresponding potential SCI targets were obtained from three traditional Chinese medicine (TCM) databases. SCI-related therapeutic target genes were obtained from the Comparative Toxicogenomics Database and the GeneCards Database. The common target genes between the JSK compounds and SCI-related therapeutic targets were screened using GO/KEGG functional enrichment and protein-protein interaction (PPI) analyses to identify hub genes and their categories of biological function. Gene expression distribution and receiver operating characteristic curve (ROC) analyses were used to identify probable SCI-related target genes. Molecular docking was used to quantify molecular interactions between target genes and the bioactive compounds of JSK.

**Results:**

A total of 183 JSK bioactive compounds and 197 target genes for the treatment of SCI were screened and assessed. The target genes were enriched primarily in drug metabolism and in inflammation-related biological processes. Ten genes with statistical significance were identified as therapeutic SCI-related target genes of JSK. Molecular docking experiments demonstrated that the proteins of these 10 genes docked with binding energies of less than −5 kcal/mol with the bioactive compounds in JSK.

**Conclusion:**

This study showed that the anti-SCI effects of JSK may be mediated through numerous bioactive components, multiple gene targets, and inflammation-related pathways and provided potential novel targets for directed therapies for treating SCI. These results provide a foundation for further experimental investigations into treatment options for SCI.

## 1. Introduction

Spinal cord injury (SCI) can result in immediate and devastating impacts on motor function and other essential physiological functions [1]. The incidence of SCI in the United States is approximately 50 per million people per year, with over 17000 new cases reported every year [2]. The World Health Organization (WHO) estimates that between 250000 and 500000 people suffer from SCI each year [3]. SCI patients who survive the initial injury usually suffer multisystem trauma concomitant with SCI, contributing to significant risks of associated complications [4]. It is reported that the lifetime cost of treating SCI in the US ranges between $1.1 million and $4.7 million, and the total cost of caring SCI patients in the US exceeds $7 billion per year [2]. Although extensively studied and the understanding of their underlying pathophysiology has improved, effective clinical treatments for SCI are limited [5]. Because of this, complementary and alternative therapies have been increasingly used for treating SCI, especially traditional Chinese medicines (TCMs) [6]. TCM has been developing for over 2000 years and has a comprehensive theoretical system for disease diagnosis and prognosis [7]. TCM provides important guidelines for the treatment of numerous complicated diseases [7].

Jisuikang (JSK) is a clinical Chinese herbal formula developed using herbs described in TCM, which has shown satisfactory efficacy in several clinical trials comparing conventional SCI treatments [8–10]. JSK could significantly improve motor function and grades of spinal injury in patients after SCI, promoting the recovery and regeneration of nerve tissues. Wang et al. found that JSK promotes nerve cell regeneration by upregulating the expression of Nogo-A that reduces the growth of axotomized neurons after SCI [11, 12]. JSK was shown to increase neurotrophic factor expression, which promotes the recovery of neurological function in SCI rats [13]. Wu et al. showed that JSK improves motor function in SCI rats by inhibiting the NgR/RhoA/ROCK signaling pathway [14], indicating the potential of JSK to regenerate nerves. However, JSK is composed of a variety of Chinese herbs [8], making it a challenge untangling the underlying molecular mechanisms of JSK in SCI recovery.

TCM therapy takes advantage of multiple herbal components, protein targets, and biological pathways, as well as their synergism, which complicates the identification of bioactive compounds, their biological targets, and their biological mechanisms [15]. Network pharmacology reveals the potential and complex interactions between compounds in Chinese herbs and targeted diseases by systematically investigating the “drug-gene-protein-disease” interaction network [16–18]. Network pharmacology integrates system biology, pharmacology, and computational biology and provides a systematic approach to studying how TCM regulates human biological networks [19]. Network pharmacology has discovered the bioactive compounds in Yinqiao and Xianlinggubao preparations that interact with candidate proteins and biological pathways for COVID-19 [20] and osteoporosis [21], respectively.

In the present study, network pharmacology was used to screen and discover the bioactive compounds and the potential pharmacological mechanisms of JSK for the treatment of SCI. Gene expression analysis and molecular docking methods were used to verify the results of the network pharmacology analysis.

## 2. Materials and Methods

### 2.1. Screening Bioactive Compounds and Targets of JSK

**Figure 1:**
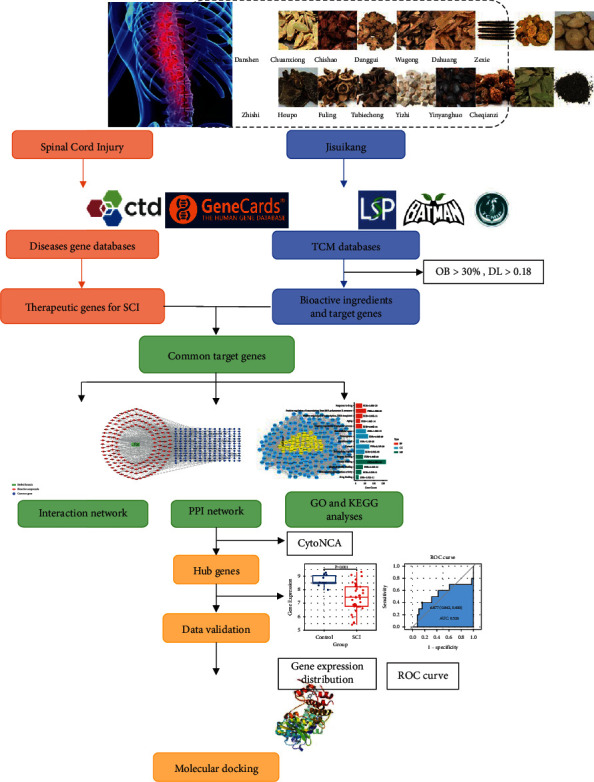
Workflow for the present study.

The workflow of this study is displayed in Figure 1. The formula of JSK consists of 16 Chinese herbs: Cheqianzi (*Plantaginis Semen*), Chishao (*Radix Paeoniae Rubra*), Chuanxiong (*Chuanxiong Rhizoma*), Dahuang (*Radix Rhei Et Rhizome*), Danggui (*Angelicae Sinensis Radix*), Danshen (*Radix Salviae*), Fuling (*Poria Cocos (Schw.) Wolf.*), Houpo (*Magnolia Officinalis Rehd Et Wils.*), Huangqi (*Hedysarum Multijugum Maxim.*), Roucongrong (*Cistanches Herba*), Tubiechong (*Eupolyphaga Seu Steleophaga*), Wugong (*Scolopendra Subspinipes Mutilans*), Yinyanghuo (*Epimrdii Herba*), Yizhi (*Alpiniae Oxyphyliae Fructus*), Zexie (*Alisma Orientale (Sam.) Juz.*), and Zhishi (*Aurantii Fructus Immaturus*). The herbal compounds were found in the Traditional Chinese Medicine Systems Pharmacology Database and Analysis Platform (TCMSP, https://old.tcmsp-e.com/tcmsp.php) [22], the Bioinformatics Analysis Tool for Molecular Mechanism of Traditional Chinese Medicine (BATMAN-TCM, http://bionet.ncpsb.org.cn/batman-tcm/) [23], and the Integrative Pharmacology-Based Research Platform of Traditional Chinese Medicine (TCMIP, http://www.tcmip.cn/TCMIP/index.php/Home/) [24]. The values for oral bioavailability (OB) and drug-likeness (DL) were set to >30% and >0.18 for screening candidate bioactive compounds [25]. All the bioactive compounds were annotated using the MOL IDs from TCMSP. Target genes of the bioactive compounds were obtained from TCMSP, and their official gene names were obtained from the UniProt Knowledgebase (UniProtKB, https://www.uniprot.org/).

### 2.2. Predicting Target Genes of SCI

The Human Gene Database (GeneCards, https://www.genecards.org/) [26] and the Comparative Toxicogenomics Database (CTD, http://ctdbase.org/) [27] were used to find potential therapeutic target genes for SCI using “spinal cord injury” as the search term.

### 2.3. Constructing the Interaction Network of JSK and Common Target Genes

Common target genes between JSK and SCI were identified using the VennDiagram package [28] in R 3.6.2 (https://www.r-project.org/). The interaction relationships between JSK bioactive compounds and the common target genes were determined, and an interaction network was constructed using Cytoscape software 3.8.2 (https://cytoscape.org/) [29].

### 2.4. Functional Enrichment Analysis

Gene Ontology (GO) enrichment analysis and the Kyoto Encyclopedia of Genes and Genomes (KEGG) signaling pathway enrichment analysis were performed to identify possible biological functions of the common target genes using the Database for Annotation, Visualization and Integrated Discovery 6.8 (DAVID 6.8, https://david.ncifcrf.gov/) [30]. The categories of cellular component, molecular function, and biological process were included in the GO terms. A false discovery rate (FDR) <0.05 was used to indicate significant differences among GO terms and KEGG pathways.

### 2.5. Protein-Protein Interaction Analysis and Identification of Hub Genes

The Search Tool for the Retrieval of Interacting Genes (STRING V11, https://www.string-db.org/) [31] was used to construct a protein-protein interaction (PPI) network of the common target genes. The minimum required interaction score was set to 0.400. The PPI network was developed using Cytoscape. The CytoNCA plugin for Cytoscape [32] was used to perform a topological network analysis that included betweenness centrality (BC), closeness centrality (CC), and degree centrality (DC) to identify hub genes among the common target genes.

### 2.6. Data Validation of Hub Genes

Gene expression distribution and receiver operating characteristic (ROC) curves of the hub genes were generated and used to investigate the association between the hub genes and prognoses in patients with SCI. The gene microarray dataset GSE151371 was downloaded from the Gene Expression Omnibus database (GEO, http://www.ncbi.nlm.nih.gov/geo/) and provided the global gene expression in peripheral white blood cells from 10 healthy individuals without a history of SCI and 38 individuals who suffered traumatic SCI. The gene expression distributions and ROC curves of the identified hub genes between healthy individuals and the SCI individuals were assessed in these two groups to determine their probability as targets for treating SCI. Hub genes that displayed statistically significant differences (a BH-adjusted *p* < 0.05) and areas under the curve (AUC) > 0.7 in the ROC analysis were considered key genes relevant to possible treatments for SCI.

### 2.7. Molecular Docking of Bioactive JSK Compounds and Proteins Encoded by Key Genes

The structure files (PDB files) of the protein products of key genes identified through gene expression distribution and ROC analyses were obtained from the Protein Data Bank (PDB, https://www.rcsb.org/) [33]. AutoDockTools V1.5.6 (http://autodock.scripps.edu/) was used to remove excess ligands, water molecules, and protein chains from the protein structures and to add hydrogens to the proteins. Bioactive compounds of JSK that were correlated with proteins encoded by the hub genes were used as molecular ligands in the docking program. The molecular structure files of bioactive JSK compounds were acquired from the PubChem database (https://pubchem.ncbi.nlm.nih.gov/), and Chem3D (https://www.codeweavers.com/) was used to construct the 3D models of the molecules (mol2 files). The PDB files of the target proteins and mol2 files of the bioactive compounds were converted into “PDBQT” formats using AutoDockTools. The docking coordinates were identified using the grid boxes in AutoDockTools. AutoDock Vina (http://vina.scripps.edu/) [34] was used to dock the molecular ligands to the corresponding proteins. The results of molecular docking experiments were analyzed and visualized using Discovery Studio 2016 and PyMOL (https://pymol.org/).

## 3. Results

### 3.1. Bioactive Compounds and Target Genes

The number of bioactive compounds found in the herbs comprising JSK is shown in Table 1. After removing duplicate compounds, a total of 183 JSK bioactive compounds were identified. There were 233 potential target genes of these bioactive compounds identified. In addition, the search of the CTD and GeneCards databases identified 16184 and 6348 SCI-related target genes, respectively.

### 3.2. Construction of “JSK-Bioactive Compounds-Common Target Genes” Interaction Network

**Figure 2:**
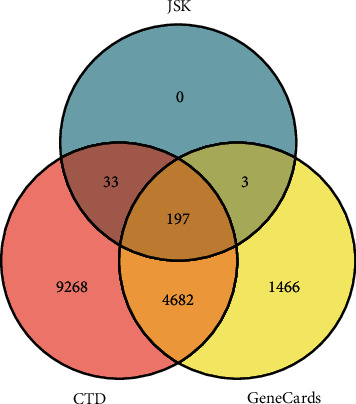
Venn diagram for JSK-related target genes and SCI-related target genes.

**Figure 3:**
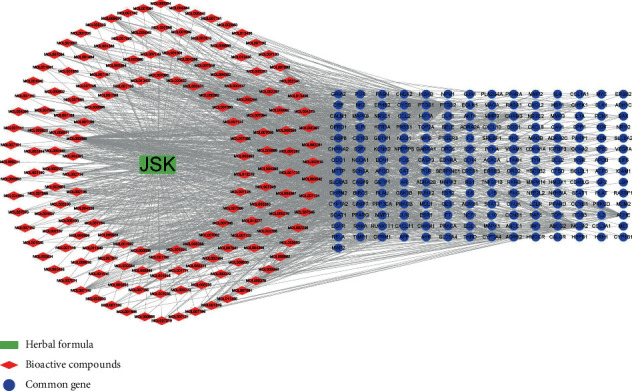
JSK-bioactive compounds-common target genes interaction network. The green rectangle represents JSK; the red diamonds represent the bioactive compounds of JSK; the blue circles represent the target genes in common between JSK and SCI.

There were 197 genes in common between JSK compound target genes and SCI-related genes (Figure 2). An interaction network analysis identified 359 nodes (1 herbal formula, 161 bioactive compounds, and 197 shared target genes) and 1632 edges (Figure 3). According to the gene count, the top 15 bioactive compounds in the network are listed in Table 2.

### 3.3. GO and KEGG Enrichment Analyses

**Figure 4:**
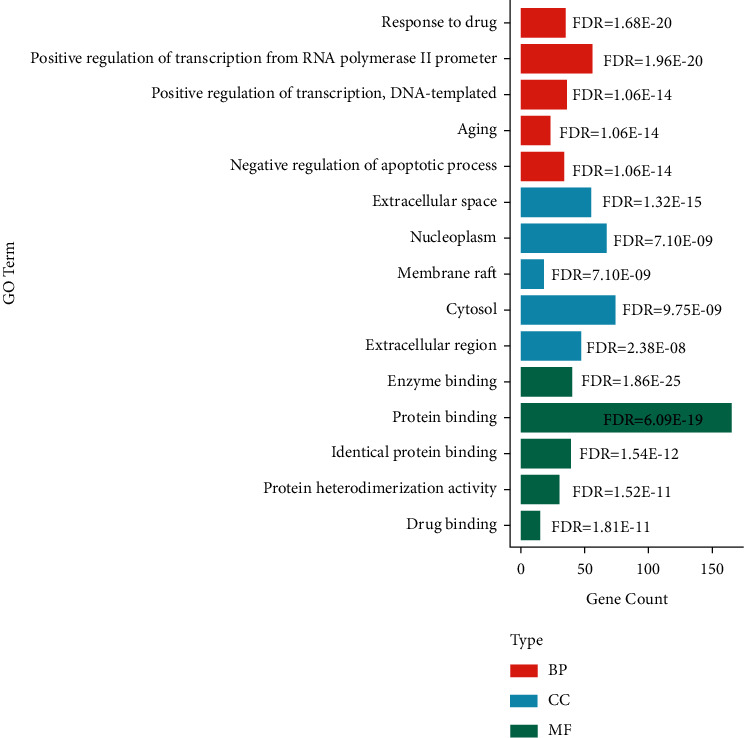
The bar plot of top five enriched GO terms of common target genes. BP: biological process; CC: cellular component; MF: molecular function; FDR: false discovery rate.

The GO terms most enriched in each category correlated to the bioactive compounds in JSK are listed in Figure 4. The top 5 enriched GO terms were enzyme binding, positive regulation of transcription from RNA polymerase II promoter, response to drug, protein binding, and extracellular space. KEGG signaling pathway analysis revealed that most of the common target genes have associations with cancer and inflammation-related pathways (Table 3).

### 3.4. PPI Analysis and Identification of Hub Genes

**Figure 5:**
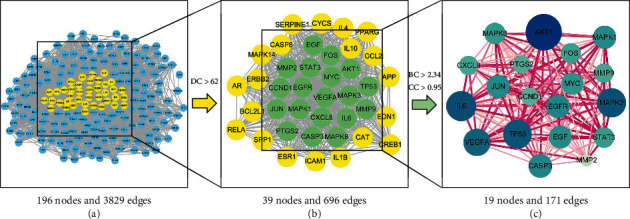
The screening process of hub genes in the PPI network.

The PPI network of common genes consisted of 197 nodes and 3829 edges. According to topological network analysis, PPI nodes are considered significant targets if the DC values were greater than twofold the median DC [35]. The median DC was set to a threshold >62 to identify significant nodes and generate a subnetwork. In the subnetwork, nodes where both BC and CC values were greater than their median (BC > 2.34, CC > 0.95) were further extracted as hub genes, and a new core network composed of hub genes was constructed (Figure 5). Important therapeutic target genes of JSK in the treatment of SCI were identified as *AKT1*, *CASP3*, *CCND1*, *CXCL8*, *EGF*, *EGFR*, *FOS*, *IL6*, *JUN*, *MAPK1*, *MAPK3*, *MAPK8*, *MMP2*, *MMP9*, *MYC*, *PTGS2*, *STAT3*, *TP53*, and *VEGFA*.

### 3.5. Hub Gene Validation

**Figure 6:**
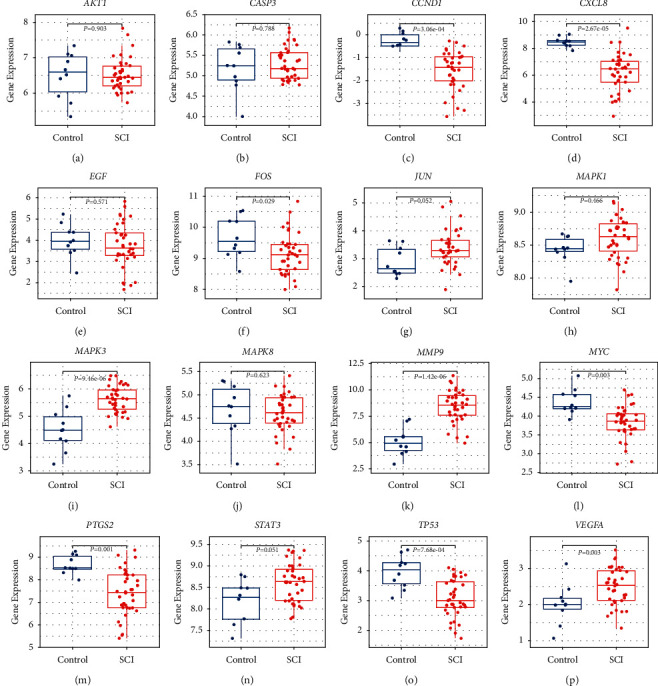
Boxplots of hub gene expression between the control group and the SCI group. (a–p) *AKT1*, *CASP3*, *CCND1*, *CXCL8*, *EGF*, *FOS*, *JUN*, *MAPK1*, *MAPK3*, *MAPK8*, *MMP9*, *MYC*, *PTGS2*, *STAT3*, *TP53*, and *VEGFA*.

**Figure 7:**
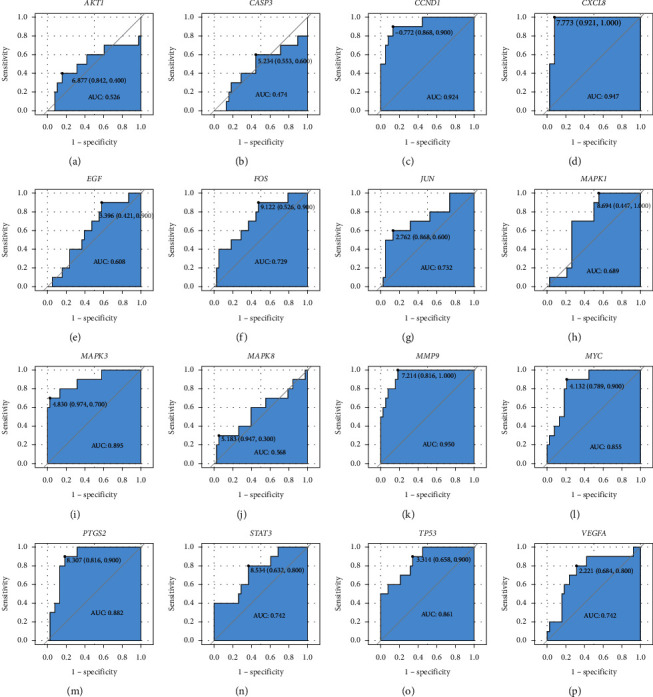
Receiver operator characteristic curves of hub genes in SCI patients. (a–p) *AKT1*, *CASP3*, *CCND1*, *CXCL8*, *EGF*, *FOS*, *JUN*, *MAPK1*, *MAPK3*, *MAPK8*, *MMP9*, *MYC*, *PTGS2*, *STAT3*, *TP53*, and *VEGFA*.

The results of the gene expression distribution of hub genes in the GSE151371 dataset (except *EGFR*, *IL6*, and *MMP2*, which were not in the GSE151371 dataset) show that 10 hub genes were downregulated in patients with SCI (Figure 6). Expression levels of *JUN*, *MAPK1*, *MAPK3*, *MMP9*, *STAT3*, and *VEGFA* were higher in the SCI group than in the control group. ROC curves and associated AUC values of these hub genes are presented in Figure 7. After combining the results of gene expression distributions and ROC curves, 10 key genes were found to have significant expression changes in SCI compared to the control: *CCND1*, *CXCL8*, *FOS*, *JUN*, *MAPK3*, *MMP9*, *MYC*, *PTGS2*, *TP53*, and *VEGFA*.

### 3.6. Results of Molecular Docking

**Figure 8:**
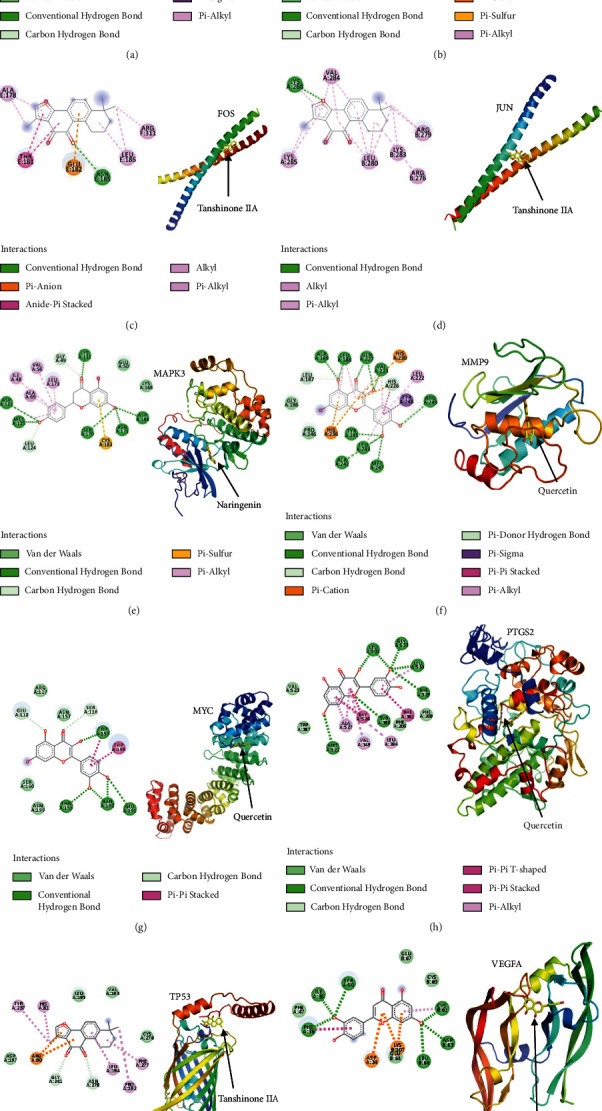
Molecular docking of protein receptors of key proteins with corresponding bioactive compounds of JSK. (a) Quercetin to CCND1; (b) quercetin to CXCL8; (c) tanshinone IIA to FOS; (d) tanshinone IIA to JUN; (e) naringenin to MAPK3; (f) quercetin to MMP9; (g) quercetin to MYC; (h) quercetin to PTGS2; (i) tanshinone IIA to TP53; (j) luteolin to VEGFA.

The protein products of the 10 key genes and the corresponding JSK compounds were chosen for molecular docking to assess their interactions. The affinity binding energies between the target proteins and the bioactive compounds were calculated (Table 4). The efficacy of molecular docking is considered to be good if the affinity binding energy is <−5.0 kcal/mol. The docking results show that quercetin, luteolin, tanshinone IIA, and naringenin had strong binding energies with their corresponding SCI proteins. The molecular dockings of the best combination of key gene proteins and their corresponding JSK compounds are presented in Figure 8.

## 4. Discussion

Individuals might face decades with permanent SCI-related disabilities. Advances in understanding the pathological mechanism of SCI have improved clinical management strategies for treating SCI, decreasing morbidity, and improving functional outcomes in patients with SCI [1]. However, randomized clinical trials have not demonstrated the efficacy and safety of conventional reparatory therapies for the recovery of motor function in SCI patients. TCM therapies, including JSK, Buyang Huanwu decoction, and Governor Vessel electroacupuncture, have provided varying degrees of therapeutic results for SCI [8, 36, 37].

The TCM theory describes the pathogenesis of SCI as a “deficiency of kidney governor, stasis of governor pulse, and dereliction of duty of cardinal command.” JSK was developed to investigate its effects in the treatment of SCI. According to TCM, the herbal compounds of JSK have the following therapeutic effects: Huangqi tonifies Qi and nourishes blood; Cheqianzi, Fuling, and Zexie help invigorate the spleen to treat diuresis; Roucongrong, Yinyanghuo, and Yizhi warm the kidney and strengthen Yang; Houpo and Zhishi regulate Qi to relieve pain; Chishao, Chuanxiong, Dahuang, Danggui, Danshen, Tubiechong, and Wugong activate blood circulation, ameliorating stasis and dredging meridians. Clinical studies have shown that JSK treatment provides significant improvement in functional recovery and the quality of life of SCI patients. However, the complex herbal composition and synergistic effects of JSK complicate the discovery of the specific mechanisms of JSK compounds for the treatment of SCI. We performed network pharmacology analysis to discover the potential bioactive compounds, biological targets, and molecular processes of JSK therapy for SCI.

### 4.1. Main Findings

The bioactive compounds in JSK are quercetin, luteolin, kaempferol, tanshinone IIA, nobiletin, baicalein, 7-O-methylisomucronulatol, anhydroicaritin, beta-sitosterol, formononetin, naringenin, C-Homoerythrinan, 1,6-didehydro-3,15,16-trimethoxy-, (3.beta.)-, miltionone I, stigmasterol, and isorhamnetin (Table 2). Quercetin protects the spinal cord and regulates secondary oxidative stress in SCI rats by inhibiting the activation of the p38MAPK/iNOS signaling pathway [38]. By activating the Nrf2/glutamate-cysteine ligase pathway, luteolin shows antioxidant, anti-inflammatory, and neuroprotective effects against SCI [39, 40]. Kaempferol reduces oxidative stress and inflammatory responses in SCI by inhibiting the MAPKs-NF-*κ*B and pyroptosis signaling pathways [41]. Tanshinone IIA improves functional recovery after SCI-induced lower urinary tract dysfunction [42]. Baicalein contributes to the functional recovery of SCI by dampening pyroptosis and alleviating endoplasmic reticulum stress-mediated apoptosis [43]. These studies support our findings of the bioactive compounds in JSK and their therapeutic role in SCI.

GO enrichment analysis revealed that the common genes were involved in regulation of transcription, enzyme binding and protein binding, response to drug, and extracellular space, indicating that the therapeutic effects of JSK may be associated with transcriptional regulation of target genes, drug metabolism, and various cell metabolic pathways. KEGG pathway analysis revealed that JSK might produce anti-SCI effects via multiple signaling pathways, including those in cancer, and the TNF, HIF-1, Toll-like receptor, and PI3K-Akt signaling pathways. The inflammatory response of nerve cells potentiates SCI damage and hinders functional recovery after SCI [44]. The PI3K-Akt pathway regulates regeneration of SCI in rats by attenuating the inflammatory response [45]. Hypoxia-inducible factor-1 (HIF-1) was found to reduce inflammation in SCI via miR-380-3p/NLRP3 and Circ 0001723 [46]. TNF was identified from a different network analysis as a major hub for inflammation following SCI [47]. In addition, certain drugs used to treat SCI are highly associated with TNF-related pathways. Overall, our results are consistent with these studies, indicating that JSK may exert anti-SCI effects through the above pathways.

We identified 19 proteins from hub genes using PPI network analysis and confirmed that 10 were associated with JSK compounds in the treatment of SCI (CCND1, CXCL8, FOS, JUN, MAPK3, MMP9, MYC, PTGS2, TP53, and VEGFA). The affinity values of all docking results were calculated to be less than −5.0 kcal/mol, indicating acceptable binding affinities between the 10 key proteins and the corresponding compounds in JSK. Our findings are consistent with numerous investigations linking proteins and pathways to SCI. Epigenetic silencing of *CCND1* in bone marrow-derived mesenchymal stem cells provides protective effects and accelerates SCI repair in rats [48]. There is evidence indicating that inhibition of the MAPK3/MAPK1 pathway could reduce inflammation and tissue injury in mice with SCI [49]. Activation of the cannabinoid-2 receptor attenuates neurological deficits in SCI by inhibiting *MMP9* expression [50]. The transcription factor p53 plays an important role in regulating the regeneration and functional recovery of axons after SCI [51]. The astrocytes with dysregulated *CXCL8* display restored sensitivity to inflammatory stimuli following spinal cord lesion, suggesting that *CXCL8* contributes to the regulation of astrocyte-mediated inflammation [52]. MiR-152 inhibits inflammatory responses and promotes the recovery of the SCI in mice through the c-jun N-terminal kinase pathway [53]. Inhibition of miR-17-5p upregulates the expression of *VEGFA* in mesenchymal stem cells (MSCs) and promotes the angiogenic ability of MSCs to repair SCI [54].

This study uncovered the multiple bioactive compounds, multiple target genes, and multiple pathways characteristics of JSK in the treatment of SCI. The above analysis results have given a general direction for research related to TCM therapy for SCI.

### 4.2. Limitations and Future Perspectives

There are limitations associated with the present study. Direct experimental validation of our findings is lacking, although our results are consistent with findings from other studies. In addition, all the components of the Chinese herbs in JSK and their SCI-related target genes may not be known, and we may have sampled only a portion of them. Both in vitro and in vivo experiments are needed to further validate our results. With the development of new analytical techniques, we anticipate that more compounds from Chinese herbs and additional therapeutic targets for SCI will be discovered and validated.

## 5. Conclusion

Based on a combination of network pharmacology approach, gene expression validation, and molecular docking, we uncovered potential genes, proteins, and molecular pathways potentially regulated by JSK compounds for the treatment of SCI. Key bioactive compounds of JSK against SCI include quercetin, luteolin, kaempferol, tanshinone IIA, 7-O-methylisomucronulatol, and nobiletin. Ten genes were identified as important therapeutic SCI-related target genes of JSK, including *CCND1*, *CXCL8*, *FOS*, *JUN*, *MAPK3*, *MMP9*, *MYC*, *PTGS2*, *TP53*, and *VEGFA*. In addition, we demonstrated that JSK might provide therapeutic effects for SCI by regulating the pathways associated with reducing inflammatory response and regenerating nerve cells, including TNF, HIF-1, Toll-like receptor, and PI3K-Akt signaling pathways. This study provides additional insights into TCM therapies for treating SCI and lays a foundation for further experimental studies.

## Figures and Tables

**Table 1:** The number of bioactive compounds in herbs from JSK.

Chinese herbs in JSK	TCMSP	BATMAN-TCM	TCMIP	Total
Cheqianzi	7	1	25	11
Chishao	13	9	16	16
Chuanxiong	6	91	34	11
Dahuang	7	30	79	13
Danggui	2	83	37	5
Danshen	58	39	34	62
Fuling	6	18	32	15
Houpo	2	42	32	6
Huangqi	16	24	24	21
Roucongrong	6	18	32	7
Tubiechong	—	1	—	1
Wugong	—	5	—	1
Yinyanghuo	21	9	17	22
Yizhi	3	19	20	4
Zexie	7	14	21	9
Zhishi	17	12	27	22

JSK: Jisuikang; TCMSP: Traditional Chinese Medicine Systems Pharmacology Database and Analysis Platform; BATMAN-TCM: Bioinformatics Analysis Tool for Molecular Mechanism of Traditional Chinese Medicine; TCMIP: Integrative Pharmacology-Based Research Platform of Traditional Chinese Medicine.

**Table 2:** The top 15 bioactive compounds in JSK.

MOL ID	Bioactive compounds	Gene count	OB (%)	DL	Corresponding herbs
MOL000098	Quercetin	119	46.43	0.28	Cheqianzi, Huangqi, Roucongrong, Yinyanghuo
MOL000006	Luteolin	46	36.16	0.25	Danshen, Yinyanghuo, Zhishi
MOL000422	Kaempferol	40	41.88	0.24	Dahuang, Huangqi, Yinyanghuo
MOL007154	Tanshinone IIA	31	49.89	0.40	Danshen
MOL000378	7-O-methylisomucronulatol	27	74.69	0.30	Huangqi
MOL005828	Nobiletin	27	61.67	0.52	Yizhi
MOL002714	Baicalein	24	33.52	0.21	Cheqianzi, Chishao
MOL004373	Anhydroicaritin	24	45.41	0.44	Yinyanghuo
MOL000358	Beta-sitosterol	23	36.91	0.75	Dahung, Danggui, Houpo, Huangqi, Roucongrong,
MOL000392	Formononetin	23	69.67	0.21	Huangqi
MOL004328	Naringenin	23	59.29	0.21	Yizhi
MOL004380	C-Homoerythrinan, 1,6-didehydro-3,15,16-trimethoxy-, (3.beta.)-	23	39.14	0.49	Yinyanghuo
MOL007119	Miltionone I	23	49.68	0.32	Danshen
MOL000449	Stigmasterol	22	43.83	0.76	Danggui, Danshen, Cheqianzi, Chishao, Yizhi
MOL000354	Isorhamnetin	22	49.60	0.31	Huangqi

OB: oral bioavailability; DL: drug-likeness.

**Table 3:** Significantly enriched KEGG signaling pathways.

Pathway ID	Pathway term	Gene count	FDR
Hsa04066	HIF-1 signaling pathway	23	2.80*E-*14
Hsa04151	PI3K-Akt signaling pathway	35	6.78*E*-11
Hsa04620	Toll-like receptor signaling pathway	21	2.06*E*-11
Hsa04668	TNF signaling pathway	27	2.14*E*-17
Hsa05133	Pertussis	17	2.66*E*-10
Hsa05142	Chagas disease (American trypanosomiasis)	20	9.23*E*-11
Hsa05145	Toxoplasmosis	21	3.54*E*-11
Hsa05152	Tuberculosis	25	1.19*E*-10
Hsa05160	Hepatitis C	22	1.19*E*-10
Hsa05161	Hepatitis B	36	2.60*E*-23
Hsa05164	Influenza A	25	9.23*E*-11
Hsa05200	Pathways in cancer	55	8.75*E*-24
Hsa05205	Proteoglycans in cancer	29	1.63*E*-12
Hsa05210	Colorectal cancer	17	2.08*E*-11
Hsa05212	Pancreatic cancer	21	1.43*E*-15
Hsa05215	Prostate cancer	23	4.22*E*-15
Hsa05219	Bladder cancer	20	1.51*E*-18
Hsa05220	Chronic myeloid leukemia	17	1.52*E*-10
Hsa05222	Small cell lung cancer	18	2.03*E*-10
Hsa05223	Non-small cell lung cancer	18	2.85*E*-13

FDR: false discovery rate.

**Table 4:** Results of molecular docking between key proteins and bioactive compounds in JSK.

Key proteins	Core compounds	Affinity (kcal/mol)	Key proteins	Core compounds	Affinity (kcal/mol)
CCND1	Luteolin	−7.2	PTGS2	7-O-methylisomucronulatol	−8.7
CCND1	Quercetin	−7.6	PTGS2	Baicalein	−9.6
CXCL8	Quercetin	−7.8	PTGS2	Beta-sitosterol	−7.2
FOS	Baicalein	−5.7	PTGS2	C-Homoerythrinan, 1,6-didehydro-3,15,16-trimethoxy-, (3.beta.)-	−6.8
FOS	Quercetin	−5.7	PTGS2	Formononetin	−9.7
FOS	Tanshinone IIA	−6.7	PTGS2	Kaempferol	−9.5
JUN	Beta-sitosterol	−6.7	PTGS2	Luteolin	−10.1
JUN	Formononetin	−5.6	PTGS2	Miltionone I	−10.1
JUN	Kaempferol	−5.6	PTGS2	Naringenin	−9.4
JUN	Luteolin	−5.9	PTGS2	Nobiletin	−8.8
JUN	Nobiletin	−5.4	PTGS2	Quercetin	−10.2
JUN	Quercetin	−5.9	PTGS2	Stigmasterol	−9.0
JUN	Tanshinone IIA	−6.8	PTGS2	Tanshinone IIA	−9.9
MAPK3	Naringenin	−8.6	TP53	Baicalein	−7.8
MMP9	Baicalein	−10.4	TP53	Luteolin	−7.5
MMP9	Luteolin	−10.7	TP53	Nobiletin	−7.1
MMP9	Nobiletin	−7.1	TP53	Quercetin	−7.6
MMP9	Quercetin	−10.7	TP53	Tanshinone IIA	−7.9
MMP9	Tanshinone IIA	−8.2	VEGFA	Baicalein	−7
MYC	Quercetin	−7.7	VEGFA	Luteolin	−7.2
MYC	Tanshinone IIA	−7.7	VEGFA	Quercetin	−6.8

## Data Availability

The data used and/or analyzed during the current study are available from the corresponding author upon reasonable request.

## Abbreviations

AKT1: AKT serine/threonine kinase 1
CASP3: Caspase 3
CCND1: Cyclin D1
CXCL8: C-X-C motif chemokine ligand 8
EGF: Epidermal growth factor
EGFR: Dermal growth factor receptor
FOS: Proto-oncogene
GO: Gene ontology
IL6: Interleukin 6
JUN: Jun proto-oncogene
KEGG: Kyoto Encyclopedia of Genes and Genomes
MAPK1: Mitogen-activated protein kinase 1
MAPK3: Mitogen-activated protein kinase 3
MAPK8: Mitogen-activated protein kinase 8
MMP2: Matrix metallopeptidase 2
MMP9: Matrix metallopeptidase 9
MYC: MYC proto-oncogene
PPI: Protein-protein interaction
PTGS2: Prostaglandin-endoperoxide synthase 2
ROC: Receiver operating characteristic curve
SCI: Spinal cord injury
STAT3: Signal transducer and activator of transcription 3
TCM: Traditional Chinese medicine
TP53: Tumor protein p53
VEGFA: Vascular endothelial growth factor A.
